# Topics of Public Interest

**Published:** 1916-02

**Authors:** 


### TOPICS OF PUBLIC INTEREST.

**Harrison Law With Reference to Osteopaths.** Treasury decision 2232 revokes 2172 and is as follows: "Osteopaths should be permitted to register and pay special tax . . . . provided they are registered as physicians or practitioners under the laws of the state." This ruling is all right if osteopaths are either recognized as legally qualified physicians or are not allowed to practice at all. But, in many states, they are given a legal status of their own without being ranked as physicians. In such states, the osteopath must, nevertheless, by the Treasury decision, fill out and swear to the same statements as regular physicians, including the statement that he is practicing medicine. In other words, the national law requires him to commit perjury under the state law. Not being an osteopath, a government official nor a lawyer, we refuse to worry about this inconsistency, but we reiterate the opinion, which is quite valueless, that the Harrison act in its entirety is unconstitutional, both because it is an invasion of state rights and because it is post facto, in applying its provisions to persons already licensed to practice medicine under a broad definition and because it affects the physician who has acquired in good faith, reasonable quantities of drugs, in a perfectly legal way, for proper use. It gets around the point of technical unconstitutionality by imposing a tax in direct violation of general principles against class legislation, and without even the excuse of raising enough money to amount to any thing beyond expenses, if indeed, it pays expenses. Whatever real good the law accomplishes could be secured by proper provision for the care and restraint of drug habitues and the ordinary legal processes directed against physicians, druggists and others, who cater to pathologic habits.

**Medical Incomes.** Richmond, Va., has about 400 physicians, 33 of whom have, according to state tax reports, incomes of

$4,000 or over (gross). Of these, 10 (8 medical men, 2 surgeons) come within the $5,000 limit. 17 have incomes between $5,000 and $10,000—12 medical men, 4 surgeons, 1 eye and ear specialist. 2 have incomes of $10,000—1 surgeon and 1 eye and ear specialist. The 3 remaining (total 32 and not 33—Ed.) are all surgeons, with incomes of $11,000; $23,000; and $42,000. According to this statement, 92% of the profession in Richmond, have incomes of less than $2,000, the limit of exemption. (Charlotte Med. Jour., Dec. 1915.)

---

**Buffalo Department of Health.**

| | 1913 | 1914 | 1916 |
|---|---|---|---|
| Population | 446,889 | 454,112 | 461,888 |
| Total births | 11,867 | 12,661 | 12,643 |
| Total birth rate per 1000 population, (excluding still births) | 26.55 | 27.77 | 27.36 |
| Total deaths | 7,043 | 7,040 | 6,853 |
| Death rate per 1000 population, (including non-residents) | 15.76 | 15.50 | 14.83 |
| Death rate per 1000 population, (excluding non-residents) | 14.72 | 14.46 | 13.99 |
| Total deaths under 1 year | 1,631 | 1,533 | 1,364 |
| Infant death rate per 1000 births | 137 | 121 | 107 |
| Total cases of typhoid fever | 502 | 417 | 248 |
| Total deaths from typhoid fever | 68 | 72 | 46 |
| Typhoid death rate per 100,000 population | 12.9 | 15.85 | 9.9 |
| Total marriages | 4,928 | 4,943 | 4,803 |

(Note: This report should be carefully studied. Such low mortality rates do not happen but are secured only by hard work. Credit is due Dr. Fronczak and his staff).

---

**Law Regarding Sale of Second-Hand Automobiles.** The seller must file immediately with the Secretary of State of N. Y., a report of date, registry number and name and address of purchaser. The latter must, within ten days, if the car has previously been licensed, file a vendee's affidavit and pay a fee of $1.00.

---

**The American Volunteer Motor Ambulance Corps** was established at the beginning of the war by Mr. Eliot. For several months it has been attached as the ambulance corps to one of the French Army Corps. It has 50 automobiles, mostly French makes, but including some Fords, 50 volunteer and 50 hired chauffeurs. Ten volunteers are needed at once. Enlistment is for three months' service. All that is necessary is good health and good temper, ability to drive a car, and submission to anti-typhoid inoculation, though some knowledge of French is desirable. This is not a medical or surgical professional service. Volunteers are expected to pay transportation and about $50 a month for extra comforts beyond regular army rations and maintenance, but it is expected that some provision can be made for those unable to afford this expense. Address Eliot Norton, Counsellor-at-Law, 2 Rector Street, N. Y. City.

---

**Reasonable Application of Law on Birth Certificates.** Many suits have been made under the state law providing that birth certificates shall be filed within five days. Several physicians have pleaded guilty and paid nominal fines—although five dollars, allowing for the fact that on the average basis of collections, it means seven or eight dollars' worth of work or a full day for the average physician, is not really nominal—after complying with the law but because the mail miscarried. Dr. Julius Richter of Buffalo defended his case. He mailed a certificate before leaving town, and his trip was unexpectedly prolonged to three weeks. Judge Hager acquitted him and established the precedent that the law should be enforced with reasonable allowance for circumstances. A verdict of this sort is welcome, not so much because of individual sparing of consequences, but it engenders a conception of the law in general as protective, as something to be respected, not as an arbitrary, unreasoning force. Birth statistics are valuable both from the standpoint of medical sociology, but for the protection of individual rights and as a social comfort. For example, we know of a person whose happiness and self-esteem has been considerably enhanced because a certain little German church kept records back in the early part of the 17th century, and of another who has been correspondingly discomfitted because certain other records were burned by the British troops during the Revolution. But it almost never happens that any practical harm arises from delay. We may allude to one other point: Physicians have been criticized for not reporting the first name of a child but have designated it as Baby ————. In many such instances, the name is not given for weeks or months, and it seems to us that the responsibility should devolve upon the parents.

---

**Drug Addicts in Buffalo.** During 1915, Dr. B. Ross Nairn examined for the Health Dept. 468 persons. 194 were adjudged insane. The crusade against narcotics and the establishment of a municipal ward for treatment, has diminished the evil considerably.

---

**About 30,000,000 Bushels of Wheat** are in storage in boats in the Buffalo harbors—enough to feed the entire population of the state of N. Y. for half a year if it were possible to use wheat products as the sole basis of nutrition, and enough for a year on a liberal allowance of actual physiologic consumption of cereal foods.

---

**The Buffalo Emergent Home Care Society,** Inc., is organized to furnish at cost nursing or domestic service in case of sickness, thus appealing to the great class that lies between charity and ample means for providing anything necessary to the comfort of the sick and their families. Four grades of help are provided: graduate nurses, non-graduate household nurses, household helpers to relieve members of the family from domestic cares, the supervising nurse, who will visit the family as needed, give advice and supervise untrained assistants. Three classes of membership are listed: active, paying $1.00 per year; fellows, including corporations, firms, lodges and other associations, paying $5.00; patrons, including individuals or aggregations of individuals, paying $500 at any time. The officers are as follows: President, Wm. E. McLennan; Vice-Presidents, Dr. James E. King, Rev. Edward C. Fellowes, Dr. Edith R. Hatch; Secretary, Dr. Franklin W. Barrows; Treasurer, Dr. A. T. Lytle. The Superintendent is Mrs Gertrude W. Boyd, R. N. Day and night calls will be received on North 1671 or Federal 47161.

---

**Dry States.** Colorado, Iowa, Washington, Oregon, Idaho, South Carolina became dry January 1. Virginia will become so November 1, making a total of 19. Nebraska, California, Michigan, South Dakota, Vermont and Alaska will vote on state prohibition this year.

---

**The Emergency Hospital of Buffalo** has undertaken a successful campaign to raise $7,000 for an X-ray equipment.

---

**Exhaust Fumes.** Dr. Hoffman, Coroner, states that six deaths occurred from the exhaust fumes of automobiles in garages, in Chicago last year. He suggests that when engines are run more than momentarily in garages, a pipe should be attached to the exhaust and conducted out doors. A Rochester physician and his chauffeur were very nearly killed by this cause a few years ago. The danger seems to lie especially with the production of carbon monoxid. A general warning should be issued and physicians should understand the nature of the case before being called in an emergency.

---

**High Mortality of Modern Warfare.** While various administrative and technical advances have reduced the mortality of the wounded in base hospitals to a low figure, recent English army reports show that 41.5% of privates struck are killed and 55% of officers. It is scarcely necessary to explain that the word "killed" does not imply instant death, and that many of the wounded die. The distinction, we understand, is according to findings at the time of collection of the wounded, but we are not certain that those dying of wounds soon after rescue are not included among the killed, and certain allusions seem to imply that the discrimination is made according to whether or not the mortally wounded reach the second line of relief stations. Statistics of 12 great battles of the Civil War give a total of 23,458 killed and 120,859 wounded, the latter including mortally wounded. Thus, the ratio of killed to the total struck was 16.26%. But the missing must also be reckoned with. Veterans of Gettysburg declared that missing meant killed for that battle, and largely for others. If all the missing of the 12 great Civil War battles are included, as killed, 33.75% of those struck were killed, but this is an overestimate, for many of the missing were captured. While exact statements are impossible, for the reason that the prime object of warfare is very far from the collection of statistics, it has been generally held, almost as an axiom of modern warfare, that the killed were about a quarter of the total struck. It has also been prophecied, with few dissenting voices, that high velocity bullets would produce, as a rule, less damage than the older ones. Of course, allowance was not made for the unprecedented use of artillery in the present war, and the equally unprecedented use of trenches with the corresponding increase of head wounds.

---

**Malpractice Insurance** has been ordered canceled in Ohio, by the State Superintendent of Insurance, on the ground that it is virtually insurance against the penalty of lack of skill or carelessness, and hence in contravention of public policy. The defense afforded by State Medical societies seems to avoid this tendency and, at the same time, protect the careful and conscientious practitioner from blackmail.

---

**Establishment of Department of Hygiene, Sanitation and Epidemiology** is announced by the H. K. Mulford Company under the executive management of Thomas W. Jackson, M. D., expert in preventive medicine, sanitation and the study and control of epidemic diseases. The most important subjects before the American people at the present time relate to the public health. Work in this field is frequently beyond the reach of the existing health and sanitary departments of the various municipalities and smaller towns, on account of limited appropriations.

The department does not propose to enter into competition with the constituted public health authorities, local, state or federal, but to aid and assist these authorities in every possible way. The work is essentially one of service and education, and will be developed along these lines. The resources and equipment of the Mulford Laboratories, chemical and bacteriological, will be utilized, thus placing at the disposal of the new department the entire laboratory facilities and expert services of the H. K. Mulford Company.

---

**Harrison Law Ruling.** Orthoform is included as a narcotic. The next step will be the inclusion of the hot water bag.

---

**Railroad Statistics.** 1914 bulletin No. 81, Bureau of Railway Economics. Mileage 387,208; revenue per mile $11,482; cost per mile $7,968. The total number of employees of all kinds was 1,695,483, who received $1,373,000,000 in salaries and wages, an average of nearly $1,000 a year apiece. 35,258,000,000 passengers were carried one mile, an average of about 352 miles per year for every inhabitant of the U. S. 9,893 persons were killed: 5,471 trespassers, 2,850 employees, 1,307 non-trespassers and 265 passengers. 79,388 persons were injured: 6,354 trespassers, 51,938 employees, 15,121 passengers. The difference between mortality and traumatisms for trespassers is noteworthy. There is an economic problem suggested by these statistics, not commonly discussed and confirmed by general impressions. Omitting travel for pleasure and that by commercial travelers so-called, the amount of time spent on business trips by the average man in business, beyond the grade of clerk, is surprising to one who, like the physician, is compelled to keep regular office hours. It is obvious that, however profitable this absentee method of conducting business may be to the individual or firm, it represents in the aggregate, an enormous economic waste, not so much in the way of direct expense for railroad fares as in loss of time and efficiency.

---

**1,000 Horses** have recently been in use by the N. Y. City Health Dept. to produce tetanus antitoxin. Much of this has been sold to Europe, at $65 per liter, a total profit of $35,000 having been made. It has been decided, however, to reduce the plant so as to provide only for the normal local demand, as commercial firms have developed means for handling the trade.

---

**Narcotic Law Ruling.** Truax, Greene & Co. of Chicago have been found guilty of selling cocaine to a licensed but not practicing physician, under a local ordinance of the city. Except to mitigate the penalty by showing absence of criminal intent, it was held not to be a defence that the clerk who made the sale had known the doctor as a practicing physician, and supposed that he wanted the drug for legitimate professional use.

---

**Witthaus Estate.** The N. Y. Academy of Medicine will receive more than $100,000 from the estate of the late Dr. R. A. Witthaus, besides his books. This fund was intended for the maintenance and extension of the library, and it was desired by Dr. Witthaus that the library be kept open Sundays, this wish having been anticipated by the Academy.

---

**Guano and the War.** Peruvian guano deposits are a very important source of the world's supply of fixed nitrogen. The enormous consumption of nitrogenous compounds in explosives threatens to exhaust the stores of the belligerent nations During the Civil War the South especially found it difficult to maintain an adequate supply of nitrites, and it was seriously proposed, as a patriotic duty, to save urine to recoup this supply. Chili saltpeter (sodium nitrate) is another important source of nitrogen for commercial and military purposes. Some years ago a novelist wrote a story based on the assumption that the extraction of nitrogen from the air for the purpose of manufacturing protein and nitrogen compounds of a commercial and military nature, on a large scale by the various nations, would so increase the oxygen content of the air as to cause a species of intoxication. It is highly improbable that such an increase of oxygen would result in such symptoms—whose influence on social and diplomatic life makes up the plot of the story—and more than an increase in the supply of water would tend to dropsy. Probably respiration would become more superficial or less frequent or both, and the only appreciable influence on human life would be from the greatly increased tendency to conflagrations of all kinds. Quite as interesting a story could have been developed on this basis instead of a physiologic speculation. However, the greatest cenceivable extraction of nitrogen from the air, would have no appreciable influence on the percentage of oxygen for some centuries. A few years after the appearance of this story, a plant was actually started at Niagara Falls for the purpose of fixing atmospheric nitrogen electrically. We understand, however, that this process is not yet on a practical scale. Thus the exhausting of natural deposits of fixed nitrogen is a very serious matter, perhaps as serious for the ultimate consideration of humanity as the more direct and obvious results of an extensive war.

---

**The Case of Lydston vs. the American Medical Association.** Briefly stated the case is as follows: Dr. Lydston, five years ago, brought suit to mandamus the state's attorney to bring quo warranto proceedings against the American Medical Association, citing that it was illegally organized in that, as a corporation of the State of Illinois and subject to its laws, it held its annual meetings outside of the state and did not elect its trustees by vote of the members but chose them indirectly by votes of persons not entitled to vote as members of the association.

Technically the case was a petition for mandamus made by Dr. Lydston to compel the state's attorney to institute quo warranto proceedings against the association and its alleged illegally elected trustees. Practically the case was and continually has been defended and fought through all courts, not by the state's attorney of Cook County, but by Frederick Z. Marx, attorney for the American Medical Association.

The defendant demurred and the demurrer was sustained by the Circuit Court. Lydston appealed to the Appellate Court, which reversed the decision of the Circuit Court, holding that it erred in sustaining the demurrer. Thereafter, **by Stipulation of parties to the suit,** the Appellate Court set aside its judgment remanding the case, overruled the demurrer and entered a final judgment awarding a peremptory writ of mandamus against the state's attorney. The defendant appealed to the Supreme Court, in spite of his consent to the attitude of the Appellate Court.

The Supreme Court held that the Appellate Court had no business to change its former judgment, in spite of the agreement of the parties, and ordered the latter court to enter its original judgment which had reversed the Circuit Court. Thereupon the Circuit Court obeyed the Appellate Court, reversing its judgment sustaining the demurrer of the state's attorney (A. M. A.) and entering a judgment which oredered the mandamus.

November 8, 1915, the Appellate Court affirmed the final judgment of the Circuit Court. December 20, the Supreme Court denied the defendant's petition for a writ of certiorari which asked the Supreme Court to hear the case on appeal. There is, therefore, nothing left for the state's attorney but to serve the writ of quo warranto against the American Medical Asociation.

The case has already been heard and decided on its merits. The original petition of Dr. Lydston for quo warranto stated the reasons therefor and these reasons were incorporated in the subsequent arguments in the different courts. The attorney for the A. M. A. (ostensibly for the state's attorney), Mr. Marx, cited precedents for his side of the case and these are overruled in the decisions. If the case goes again before these courts the judgments will be reaffirmed because the questions at issue have already been argued before the courts and have been the basis of the decisions already handed down.

From the above it is evident that the Association must sooner or later reorganize along democratic lines. What these lines shall be depends upon the members into whose hands the courts have practically delivered the Association.

Quoted in full from the Illinois Medical Journal, Jan. 1916, the official organ of Illinois State Medical Society.

